# Epidemiological patterns and risk profiling of hospital acquired venous thromboembolism in a tertiary medical center in eastern China: a retrospective cohort study (2023–2024)

**DOI:** 10.3389/fcvm.2026.1707402

**Published:** 2026-07-01

**Authors:** Zheng Yang, Jie Qin

**Affiliations:** Department of Quality Improvement, Taizhou Hospital of Zhejiang Province, Wenzhou Medical University, Linhai, China

**Keywords:** deep vein thrombosis, hospital acquired venous thromboembolism, mortality, prophylaxis, pulmonary embolism, risk factor, surgical risk stratification

## Abstract

**Objectives:**

To investigate the epidemiological patterns, clinical characteristics, and risk factors of hospital acquired venous thromboembolism (HA-VTE) in a multi-campus medical center over a two-year period.

**Methods:**

A retrospective analysis were performed on patients diagnosed with HA-VTE at a tertiary medical center from January 1, 2023 to December 31, 2024. Parameters potentially affecting the occurrence of HA-VTE were analyzed using SPSS 27.0. The comparison group for risk factor analysis comprised all hospitalized patients without VTE during the same period.

**Results:**

A total of 393 inpatients met the criteria for HA-VTE. Among these, 308 presented with isolated hospital acquired deep vein thrombosis (HA-DVT), 61 with isolated hospital acquired pulmonary embolism (HA-PE), and 24 with concomitant HA-DVT and HA-PE. Risk factor analyses showed statistically higher incidence in patients with older age, malignancy, trauma, ICU stay, and major surgery. Patients with HA-VTE had longer hospital stays and higher mortality rates. The departments with the highest incidence rate were Neurosurgery, Intensive Care Unit (ICU), and Orthopedics.

**Conclusion:**

This study highlights a strong association between specific surgical clusters (prolonged mechanical ventilation, central venous access, and major orthopedic/neurosurgical procedures) and HA-VTE incidence. The interplay of endothelial injury, stasis, and hypercoagulability underscores the need for tailored prophylaxis strategies. Patients with death related to venous embolism have the characteristics of advanced age, multiple comorbidities, more iatrogenic interventions, and inadequate infection control. Clinicians need to strengthen VTE risk assessment and stratified management, with particular attention to postoperative, infected, and critically ill patients, to reduce mortality.

## 1 Introduction

Hospital-acquired venous thromboembolism (HA-VTE), encompassing hospital-acquired deep vein thrombosis (DVT) and hospital-acquired pulmonary embolism (PE), continues to be a leading cause of preventable morbidity and mortality among hospitalized patients worldwide (1). Despite advances in preventive measures, HA-VTE remains a critical patient safety issue, contributing to extended hospital stays, increased healthcare costs, and mortality rates as high as 30% in untreated cases. Current epidemiological data, primarily from Western populations, indicate that HA-VTE constitutes about 50%–70% of all VTE cases in tertiary care settings, with risk being influenced by patient-specific factors (e.g., age, immobility, malignancy) and institutional protocols (2, 3). The pathogenesis of VTE is classically attributed to Virchow's triad: endothelial injury, venous stasis, and hypercoagulability. Specific surgical procedures and traumatic injuries disproportionately disrupt these elements. For instance, major orthopedic surgeries—particularly total hip or knee arthroplasty and hip fracture repair—are associated with a VTE risk as high as 40%–60% in the absence of prophylaxis, due to prolonged immobility, direct vascular manipulation, and intraoperative bone marrow debris release (4). Neurosurgical procedures, including craniotomy for tumor resection or intracranial hematoma evacuation, similarly confer elevated risk through extended operative times, postoperative immobilization, and the release of brain-derived tissue factor (5). Furthermore, abdominal and pelvic cancer surgeries (e.g., pancreaticoduodenectomy, hysterectomy with lymphadenectomy) are complicated by VTE in up to 30% of cases, driven by malignancy-associated hypercoagulability and extensive pelvic venous manipulation (6). Trauma patients, especially those with spinal cord injury, pelvic fractures, or multiple long-bone fractures, exhibit VTE rates exceeding 50% without prophylaxis due to systemic inflammation, endothelial disruption, and prolonged recumbency (7). Significant gaps exist in understanding region-specific patterns, particularly in healthcare systems with diverse resource allocations and patient demographics. Existing risk assessment models, such as the Padua and Caprini scores, show inconsistent predictive accuracy across different populations, highlighting the necessity for risk stratification tools that are tailored to specific contexts (8). This retrospective cohort study aims to outline the epidemiological features, temporal patterns, and risk profiles of HA-VTE at a high-volume tertiary medical center from 2023 to 2024. By analyzing a large, diverse inpatient population, we seek to identify modifiable risk factors, assess adherence to thromboprophylaxis guidelines, and evaluate the real-world effectiveness of established risk prediction models, thereby guiding targeted prevention strategies and resource allocation, and decrease the impact of HA-VTE in settings with limited resources, where delayed diagnosis and substandard prophylaxis are common challenges.

## 2 Methods and materials

### 2.1 Study design and population

This retrospective cohort study was conducted at a multi-campus tertiary medical center (Hospitals A, B, and C) in Eastern China. Data from all hospitalized patients aged ≥13 years (excluding day ward patients) between January 1, 2023, and December 31, 2024, were extracted from electronic medical records. The study was approved by the institutional review board, and the requirement for written informed consent was waived due to the retrospective nature of the analysis.

### 2.2 Inclusion and exclusion criteria

Patients were included if they had a confirmed diagnosis of VTE (DVT or PE) during hospitalization, based on imaging evidence (Doppler ultrasound for DVT; computed tomography pulmonary angiography for PE). HA-VTE was defined as VTE diagnosed after hospital admission and not present at the time of admission, with a minimum interval of 24 h between admission and diagnosis to exclude community-acquired events. Cases with unclear onset timing were excluded after independent review by two investigators. Both symptomatic events (triggered by clinical suspicion) and asymptomatic events (detected by screening ultrasound in high-risk immobilized patients) were included. Duplicate events were identified using unique patient identifiers and the earliest event per hospitalization was retained. Exclusion criteria were: age <13 years, admission to day wards, and presence of VTE at admission (community-acquired VTE), and recurrent VTE admissions without new events.

### 2.3 Data collection

Variables collected included: demographics (age, sex), length of hospital stay, comorbidities (hypertension, diabetes, malignancy, chronic kidney disease, etc.), surgical procedures (type and date), VTE subtype (isolated DVT, isolated PE, concomitant DVT/PE), mechanical ventilation duration (≥96 h vs. < 96 h), central venous catheterization, pharmacological and mechanical prophylaxis use, and clinical outcomes (mortality, recurrent VTE, ICU admission, hospitalization costs). For deceased patients, additional data on direct cause of death and infection-related complications were recorded.

### 2.4 Statistical analysis

Descriptive statistics were used to summarize patient characteristics. Continuous variables were expressed as median (interquartile range) or mean ± standard deviation as appropriate. Categorical variables were compared using chi-square or Fisher's exact tests. To identify independent predictors of HA-VTE, multivariate logistic regression was performed comparing the 393 HA-VTE cases with all hospitalized patients without VTE during the same period (*n* = 449,634). Candidate variables included age (≥65 years vs. < 65 years), sex, immobilization >72 h, major surgery (any procedure requiring general or neuraxial anesthesia), active malignancy, central venous catheterization, ICU stay, trauma, and mechanical ventilation ≥96 h. Variables were selected based on clinical relevance and univariate association (*p* < 0.10). Missing data were handled by complete-case analysis, as missingness was <5% for all variables. Collinearity was assessed using variance inflation factors (VIF), with no evidence of significant collinearity (all VIF <2.5). Results are presented as adjusted odds ratios (OR) with 95% confidence intervals (CI) and two-tailed *p*-values. All analyses were conducted using SPSS version 27.0 (IBM Corp., Armonk, NY, USA). A two-tailed *p*-value <0.05 was considered statistically significant.

## 3 Results

### 3.1 Hospital discharge characteristics

Table 1 summarizes annual discharge numbers and gender distribution across the three hospitals from 2023 to 2024. Hospital A had a significantly higher proportion of male discharges (51.51% over two years) compared to Hospital B (50.49%) (*χ*^2^ = 42.32, df = 1, *p* < 0.001). Hospital C exclusively served female patients (100%), reflecting its specialization in obstetrics and gynecology. Gender proportions remained stable across the two years in both Hospital A (*p* = 0.729) and Hospital B (*p* = 1.000) (Table 1).

**Table 1:** Annual discharge numbers and gender distribution by hospital from 2023 to 2024.

Hospital	Year	Total Discharges	Male (n)	Male (%)	Female (n)	Female (%)
A	2023	151,452	77,979	51.49%	73,473	48.51%
A	2024	160,196	82,557	51.53%	77,639	48.47%
B	2023	65,762	33,205	50.49%	32,557	49.51%
B	2024	69,582	35,131	50.49%	34,451	49.51%
C	2023	1,429	0	0.00%	1,429	100.00%
C	2024	1,606	0	0.00%	1,606	100.00%
Total	2023–2024	450,027	228,872	50.86%	221,155	49.14%

### 3.2 Prophylaxis implementation rates

Among patients assessed as high-risk for VTE with low bleeding risk, the implementation rate of pharmacological prophylaxis was 80% in Hospital C, but only 43.28% in Hospital A (*p* < 0.01), indicating significant gaps in guideline adherence. The risk assessment model used varied by department: some used the Caprini score (surgical patients) or Padua score (medical patients), but implementation was not systematic across all units. Low bleeding risk was defined as absence of active bleeding, no major bleeding history within 4 weeks, platelet count >50  ×  10⁹/L, and no concurrent antiplatelet therapy. Reasons for withholding prophylaxis, extracted from medical records, included perceived bleeding risk (54% of non-adherent cases), patient refusal (12%), and omitted prescription (34%). Mechanical prophylaxis (intermittent pneumatic compression or graduated compression stockings) was used alone in 28% of high-risk patients and in combination with pharmacological prophylaxis in only 18% of cases.

### 3.3 Demographic characteristics of 393 HA-VTE patients

**Figure 1:**
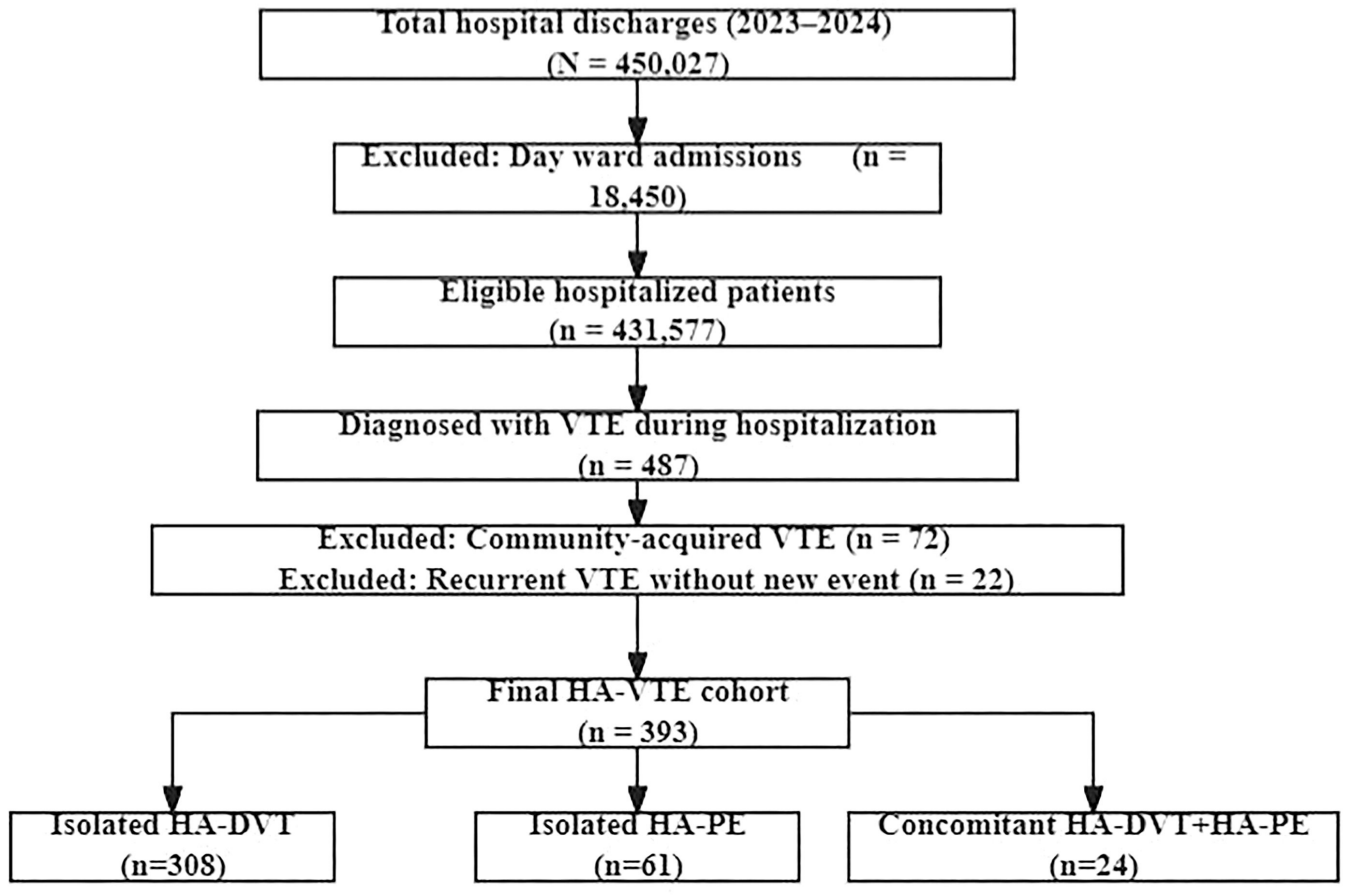
Screening flow chart of HA-VTE. Flow diagram showing identification of 393 HA-VTE cases from 450,027 hospital discharges between 2023 and 2024. HA-VTE, hospital-acquired venous thromboembolism; HA-DVT, hospital-acquired deep vein thrombosis; HA-PE, hospital-acquired pulmonary embolism.

**Figure 2:**
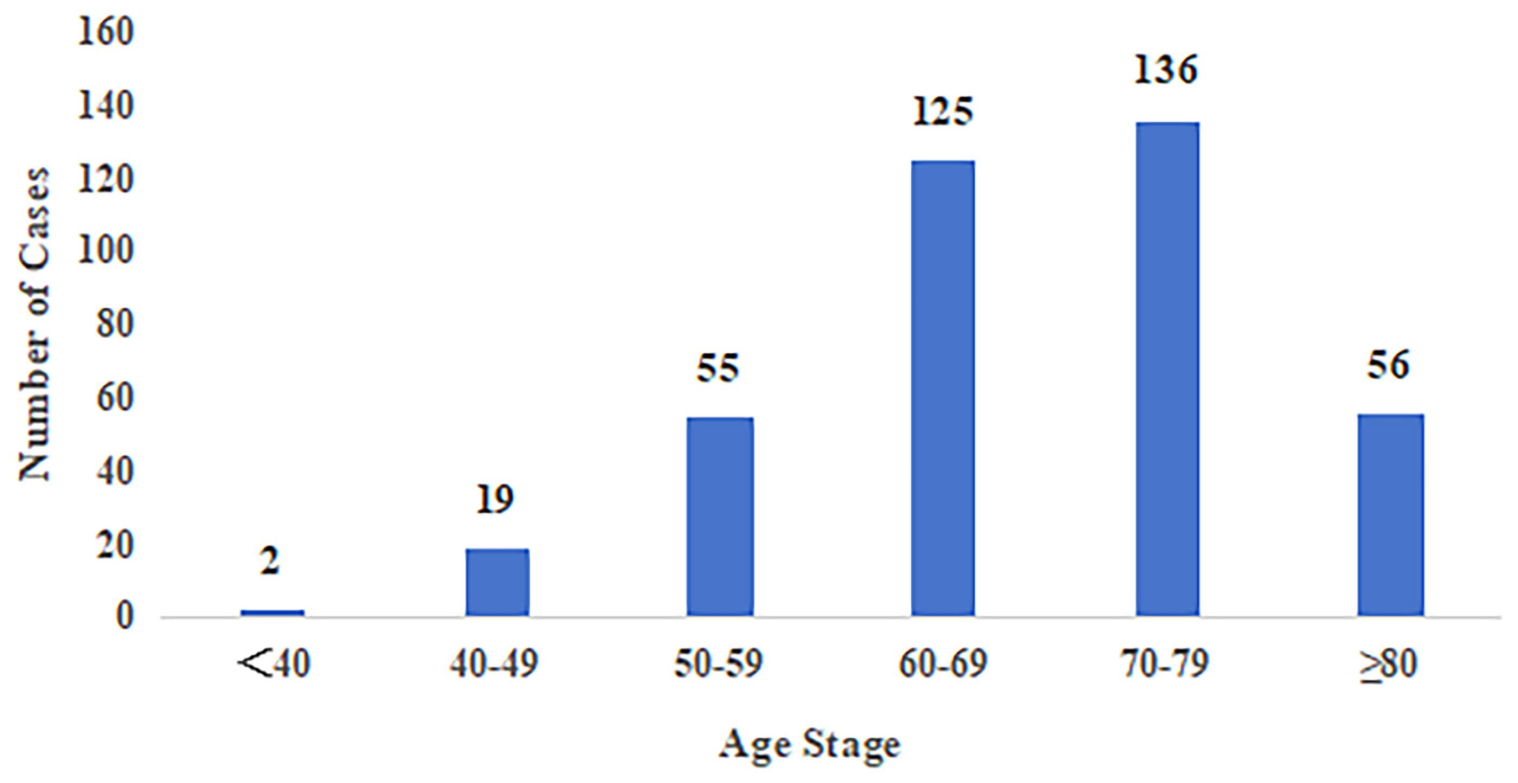
Distribution of HA-VTE patients across different age stages (*n* = 393). Bar chart showing the number of HA-VTE cases in each age group (<40, 40-49, 50-59, 60-69, 70-79, ≥80 years). The highest number of cases occurred in the 70-79 years age group.

A total of 393 inpatients met the HA-VTE criteria (Figure 1). The overall incidence of HA-VTE was 0.87 per 1,000 admissions (393/450,027). The median age was 69 years (IQR: 61–75), with 80.7% aged ≥60 years (Figure 2). Females accounted for 52.1% of cases. The median hospital stay was 20 days (IQR: 21–28), with longer stays correlated with higher VTE risk (OR = 2.1, *p* < 0.01).

### 3.4 Clinical features of 393 HA-VTE

Among the 393 HA-VTE patients, lower extremity HA-DVT accounted for 74.6%, HA-PE for 21.6%, and upper extremity HA-DVT for 6.1%. Isolated HA-DVT was the predominant subtype (*n* = 308, 78.4%), followed by isolated HA-PE (*n* = 61, 15.5%) and HA-PE with concomitant HA-DVT (*n* = 24, 6.1%). Common comorbidities included hypertension (43.0%), malignancy (23.7%), diabetes (19.6%), and chronic kidney disease (8.9%). Overall, 59.5% of HA-VTE cases occurred in the postoperative setting, predominantly following neurosurgery (24.4%), orthopedic surgery (11.96%), and cardiothoracic surgery (6.1%).

### 3.5 Independent risk factors for HA-VTE

Multivariate logistic regression (comparing 393 HA-VTE cases with 449,634 non-VTE hospitalizations) identified the following independent predictors of HA-VTE (*p* < 0.05): immobilization >72 h (OR = 3.4, 95% CI: 2.7–4.3, *p* < 0.001), major surgery (OR = 2.9, 95% CI: 2.3–3.7, *p* < 0.001), active malignancy (OR = 2.7, 95% CI: 2.1–3.5, *p* < 0.001), central venous catheterization (OR = 2.2, 95% CI: 1.7–2.8, *p* < 0.001), and age ≥65 years (OR = 1.8, 95% CI: 1.4–2.3, *p* < 0.001). Full model results including all candidate variables are provided in Supplementary Table S1.

### 3.6 Clinical outcomes

Overall mortality among HA-VTE was 1.53% (6/393), with three cases directly attributed to PE. Recurrent VTE (defined as new DVT in a different extremity or new PE during the same hospitalization or after readmission within 30 days) occurred in 22. 1% of patients, and 23. 2% required ICU admission. HA-VTE extended hospitalization by a median of 8 days (95% CI: 5–11) and increased costs by 42% (*p* < 0.001).

### 3.7 High-risk surgical procedures and mechanistic links

#### 3.7.1 Precedure frequency and clusters

Retrospective analysis of the 393 HA-VTE cases revealed distinct high-risk categories. Prolonged mechanical ventilation (≥96 h) was recorded in 121 cases (30.8%). Central venous catheterization—internal jugular (*n* = 68, 17.3%) and femoral (*n* = 45, 11.5%)- was also common. Orthopedic surgeries (total hip replacement, femoral fracture open reduction) occurred in 47 cases (12.0%); neurosurgical procedures (intracranial hematoma evacuation, decompressive craniectomy, dural patch repair) in 99 cases (25.2%). Abdominal/pelvic oncologic resections (e.g., pancreaticoduodenectomy, hysterectomy with lymphadenectomy) were noted in 43 cases (10.9%). Procedural combinations markedly elevated risk: decompressive craniectomy with prolonged mechanical ventilation conferred a 3.2-fold increased thrombosis risk (*p* < 0.01), and hip replacement combined with femoral vein catheterization correlated with a 2.8-fold rise in DVT incidence.

#### 3.7.2 Mechanistic data (exploratory analysis)

In an exploratory, non-predefined analysis of a subset of patients, we directly measured several biomarkers to explore pathogenesis. Among post-neurosurgery patients (*n* = 50 consecutively sampled), 78% had elevated D-dimer (>2,000 ng/mL) and fibrinogen (>450 mg/dL) levels, indicating a hypercoagulable state. Blood samples were collected within 24 h of VTE diagnosis. Furthermore, flow cytometry analysis of plasma samples revealed that tumor-derived microparticles expressing tissue factor (TF) and cancer procoagulant (CP) were detected in 41% of oncologic surgery patients (*n* = 18/44 tested). TF + and CP + positivity was defined as levels exceeding two standard deviations above the mean of healthy controls (*n* = 20). These findings support direct endothelial injury from invasive procedures (e.g., catheterization, neurosurgery) as a trigger for the coagulation cascade via TF release, and confirm a cancer-related prothrombotic state in a substantial subset of patients. Given the exploratory nature and small sample sizes, these results are hypothesis-generating and require validation in larger prospective studies.

### 3.8 Characteristics of fatal HA-VTE cases

Six patients died during the study period (Table 2). The mean age was 79.9 ± 10.7 years, and 83.3% were male. Infection-related complications (sepsis, septic shock, or COVID-19) were present in five cases. All six patients had multiple organ failure (respiratory and renal failure being most common). Three deaths were directly attributed to PE, while the other three died from underlying disease complications. All fatal cases had prolonged immobilization (median 21 days, range 11–84 days) and had received suboptimal anticoagulation prophylaxis. Due to the small number of events (*n* = 6), these data are presented descriptively and do not support robust statistical inference regarding mortality predictors.

**Table 2:** Demographic and clinical characteristics of death cases with HA- VTE in A Two-year period.

Variables	Case 1	Case 2	Case 3	Case 4	Case 5	Case 6
Sex	Male	Male	Male	Female	Male	Male
Age (years)	63	66	80	85	86	94
Hospital stay (days)	18	84	11	22	12	96
Primary Diagnosis	Paralytic ileus	Intracranial hemorrhage (non-traumatic)	Coronary atherosclerotic heart disease	Femoral neck fracture	Cerebral infarction	Cerebral contusion
Secondary Diagnosis	Diffuse peritonitis; Acute respiratory failure; Sepsis; Septic shock; Hepatic insufficiency; Acute renal insufficiency; Multiple organ dysfunction syndrome; Bacterial pneumonia; Pulmonary fungal infection; Pleural effusion; Ascites; Lower limb muscular venous thrombosis; COVID-19	Subarachnoid hemorrhage; Brain herniation; Type I respiratory failure; Severe pneumonia; Shock; Right lung malignancy; Secondary pleural malignancy; Viral pneumonia; Invasive pulmonary aspergillosis; COVID-19; Sepsis;Lower limb deep vein thrombosis; Post-chemotherapy myelosuppression	Post-coronary stent implantation status; High-grade atrioventricular block; Third-degree atrioventricular block; Cardiogenic syncope; Carotid artery occlusion; Essential hypertension; Type 2 diabetes mellitus; Lacunar infarction; Cardiac arrest；Pulmonary embolism	Pneumonia; Acute respiratory failure; Intertrochanteric fracture; Septic shock; Pulmonary mass; Hypostatic pneumonia; Lacunar infarction; Cerebral infarction; Lower limb muscular venous thrombosis; Pulmonary embolism	Cardiac arrest; Pulmonary embolism; Respiratory failure; Acute coronary syndrome; Acute exacerbation of chronic obstructive pulmonary disease; Lower limb deep vein thrombosis; Atrial fibrillation; Pulmonary mass	Traumatic subdural hemorrhage; Respiratory failure; Traumatic subarachnoid hemorrhage; Traumatic epidural hematoma; Pneumonia; Old pulmonary tuberculosis; Atrial fibrillation; Lower limb muscular venous thrombosis; Nutritional risk
Surgical/Procedural Interventions	2023-06-26: Temporary ileostomy; 2023-06-26: Laparoscopic adhesiolysis; 2023-06-26: Mechanical ventilation [≥96 h]; 2023-06-27: CRRT; 2023-06-26: Cardiac output monitoring (PiCCO); 2023-06-29: Bronchoscopy with bronchoalveolar lavage; 2023-06-20: Intestinal decompression tube placement; 2023-06-26: Internal jugular vein catheterization	2023-10-13: Mechanical ventilation [≥96 h]; 2023-08-16: Transcatheter bronchial artery embolization; 2023-09-14: Radiotherapy; 2023-08-16: Bronchial artery angiography; 2023-08-22: DBAL; 2023-08-22: Bronchoscopic biopsy; 2023-10-13: Other tracheal lavage; 2023-10-13: Central venous catheterization under guidance	2024-12-11: Dual-chamber permanent pacemaker implantation; 2024-12-06: Percutaneous coronary drug-coated balloon angioplasty; 2024-12-06: IVUS; 2024-12-06: Percutaneous coronary balloon angioplasty; 2024-12-06: Single-vessel intervention; 2024-12-06: Single-catheter coronary angiography; 2024-12-06: Left heart catheterization; 2024-12-03: Single-catheter coronary angiography	2023-01-30: Artificial femoral head replacement; 2023-01-30: Open reduction and internal fixation of femoral fracture; 2023-02-02: Femoral vein catheterization	2023-03-06: Endotracheal intubation; 2023-03-06: Cardiopulmonary resuscitation	2023-10-22: Mechanical ventilation [≥96 h]; 2023-11-03: Bronchoscopy; 2023-11-08: DBAL
VTE type	HA-DVT	HA-DVT	HA-PE	HA-PE + HA-DVT	HA-PE + HA-DVT	HA-DVT
Direct Causeof Death	Not PE	Not PE	PE	PE	PE	Not PE

All six patients had prolonged immobilization (median 21 days, range 11–84 days). Five of six had infection-related complications (sepsis, septic shock, or COVID-19). Three deaths were directly attributed to PE; the other three died from underlying disease complications.

### 3.9 Departmental distribution of HA-VTE

**Figure 3:**
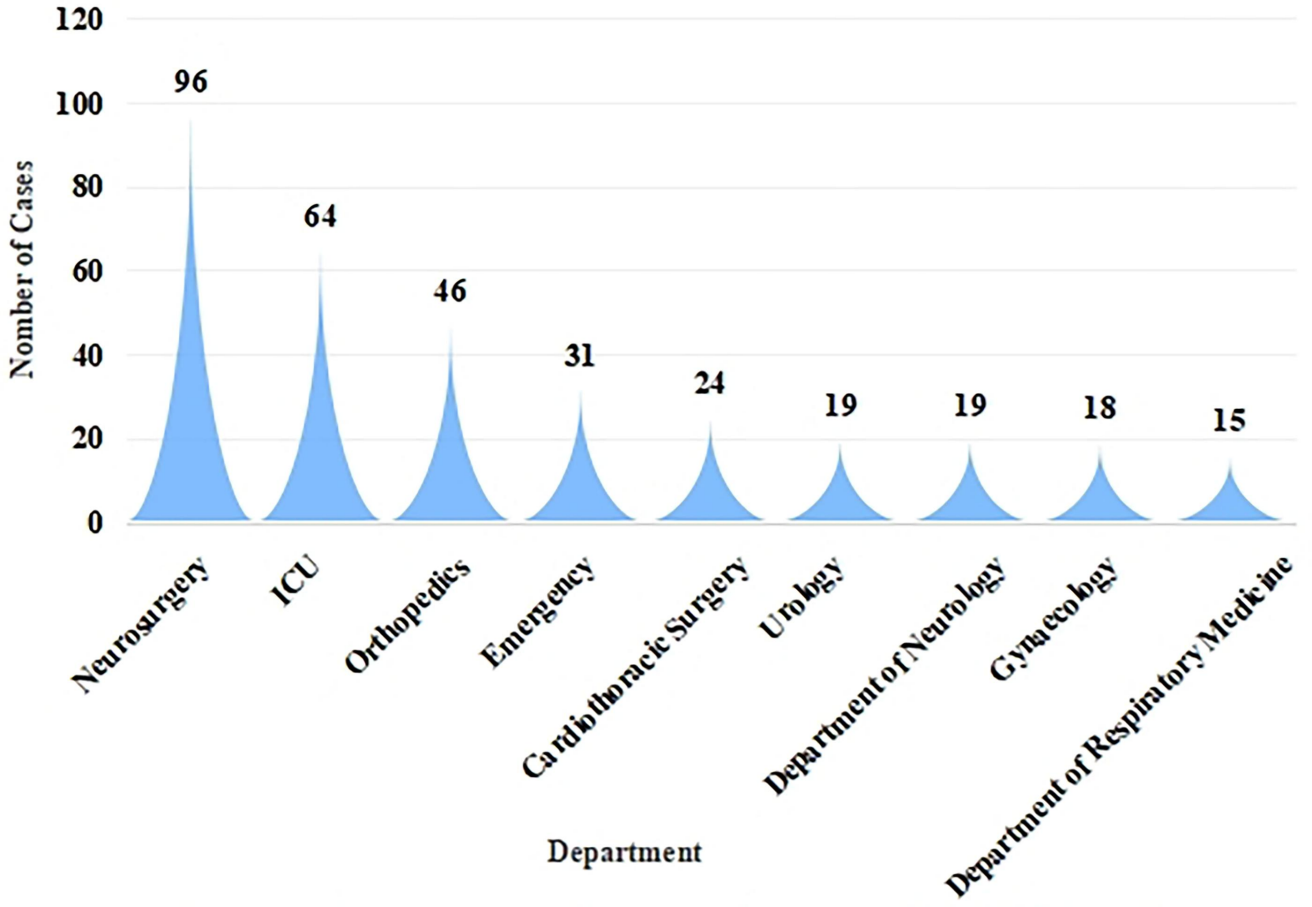
Department distribution of discharges with HA-VTE (*n* = 393). Bar chart showing the number of HA-VTE cases by clinical department. Neurosurgery had the highest number (*n* = 96, 24.4%), followed by Intensive Care Unit (*n* = 64, 16.3%) and Orthopedics (*n* = 46, 11.7%). These are absolute case counts, not incidence rates, as department-specific denominators were not available.

Significant heterogeneity was observed across discharge departments (Figure 3). Neurology Surgery had the highest number of HA-VTE (*n* = 96, 24.4%), followed by the Intensive Care Unit (*n* = 64, 16.3%) and Orthopedic departments (*n* = 46, 11.7%). Other departments with notable case counts included Emergency (mainly surgical inpatients, *n* = 31), Cardiothoracic Surgery (*n* = 24), and Hepatobiliary Surgery (*n* = 13). Non-surgical departments such as Respiratory Medicine (*n* = 15) and Oncology (*n* = 5) had lower but non-negligible counts. Notably, surgical specialties dominated the high-risk categories. Because department-specific denominators (e.g., number of admissions or patient-days) were not available, incidence rates per 1,000 admissions could not be calculated; thus, these data reflect absolute case counts and should be interpreted as indicators of disease burden rather than true risk.

## 4 Discussion

This retrospective cohort study of 393 HA-VTE cases in a large tertiary medical center in Eastern China provides contemporary insights into the epidemiological patterns, risk factors, and clinical outcomes of hospital-acquired venous thromboembolism. Our findings confirm that HA-VTE remains a substantial burden, disproportionately affecting elderly, critically ill, and surgical patients, with notable gaps in prophylaxis adherence.

### 4.1 High-risk departments and comparison with previous studies

The highest numbers of HA-VTE cases were observed in Neurosurgery (24.4%), ICU (16.3%), and Orthopedics (11.7%). These findings align with those of Li et al. (1) and Crespi et al. (4), who reported that neurosurgical and orthopedic procedures confer a 20%–50% baseline VTE risk in the absence of prophylaxis. The particularly high burden in our ICU cohort (*n* = 64) is consistent with recent work by Havlicek et al. (9), who demonstrated that invasive mechanical ventilation ≥96 h increases VTE risk nearly fourfold, and with Khan et al. (10), who identified ICU-specific factors such as central venous catheters and prolonged sedation as independent contributors. Notably, our departmental distribution mirrors that reported by Fischer et al. (11) in a North American spine surgery cohort, suggesting that surgical VTE risk patterns may be broadly generalizable across diverse healthcare systems. However, without department-specific denominator data, we cannot determine whether Neurosurgery had the highest incidence or simply the largest volume of high-risk patients. Future studies should report incidence per 1,000 admissions or per 10,000 patient-days to enable valid cross-departmental comparisons.

### 4.2 Risk factors and independent predictors

Multivariate analysis identified immobilization >72 h (OR = 3.4), major surgery (OR = 2.9), active malignancy (OR = 2.7), central venous catheterization (OR = 2.2), and age ≥65 years (OR = 1.8) as independent predictors. These findings are highly consistent with the systematic review by Bakhsh (8) and the large-scale electronic health record analysis by Neeman et al. (2), both of which confirmed malignancy and central venous access as dominant risk factors. However, the magnitude of the immobilization effect in our cohort (OR = 3.4) exceeds that reported in some Western studies (typically OR 1.8–2.5) (3, 12), possibly reflecting longer durations of postoperative bed rest in our setting due to cultural practices or staffing constraints. This discrepancy suggests that context-specific risk weighting may be necessary when applying Western-derived risk models to Eastern populations. Comparison with large real-world registries such as RIETE further supports the generalizability of key risk factors; for instance, Mastroiacovo et al. demonstrated that prolonged hospitalization is common in acute PE and associated with adverse outcomes, consistent with our finding of extended stays (13).

### 4.3 Prophylaxis gaps and mortality

The low pharmacological prophylaxis implementation rate in high-risk patients at Hospital A (43.28%) compared to Hospital C (80%) is alarming and likely contributed to the observed HA-VTE burden. Similar gaps have been reported in other settings: Watt et al. (14) found that junior doctors frequently omit thromboprophylaxis due to knowledge deficits, while Oh et al. (15) documented suboptimal nurse-led risk assessment in South Korea. Importantly, the 1.53% overall mortality in our HA-VTE cohort—with half directly attributable to PE—underscores the clinical consequences of under-prophylaxis. This is comparable to the 2.1% hospital-acquired VTE mortality reported by Shao et al. (3) in a Chinese multicenter study, but lower than the 5%–10% often cited in historical Western cohorts (16), possibly reflecting improved rescue therapies. Recent RIETE registry analyses have highlighted the importance of dynamic risk assessment; for example, Siniscalchi et al. showed that statin use was associated with lower 30-day mortality in acute PE, while a subsequent study found that low LDL-cholesterol levels increased bleeding risk during anticoagulation, emphasizing the need to balance thrombotic and hemorrhagic risks (17, 18).

### 4.4 Predominance of isolated DVT and diagnostic implications

Isolated HA-DVT accounted for 78.4% of cases, a proportion higher than in some Western series (typically 50%–70%) (2, 19). This may reflect our institution's routine use of lower-extremity Doppler ultrasound screening in immobilized patients—a practice recommended by Tran et al. (20) and Abunimer et al. (21)—coupled with possible underdiagnosis of PE due to overlapping symptoms with other critical illnesses. The low rate of concomitant DVT and PE (6.1%) suggests that either thrombus migration is infrequent or that PTE is missed without systematic screening protocols (16, 21). These findings advocate for aggressive DVT surveillance in high-risk immobilized patients and protocolized PTE evaluation (D-dimer followed by CT pulmonary angiography) when respiratory symptoms arise, as proposed by Hou et al. (22) and Oh et al. (16). In line with this, Expósito-Ruiz et al. showed that most postoperative VTE events occur within the first two weeks after surgery, supporting early and systematic surveillance (23).

### 4.5 Mechanistic insights and biomarker findings (exploratory)

Our direct measurement of D-dimer, fibrinogen, TF-bearing microparticles, and CP in a subset of patients provides novel mechanistic data from an Eastern population. The detection of TF + and CP + microparticles in 41% of oncology surgery patients extends the experimental observations of cancer-associated hypercoagulability (6) to the clinical surgical setting. Furthermore, the association of prolonged mechanical ventilation (≥96 h) with a 3.2-fold increased risk in neurosurgical patients supports the call by Lewis et al. (24) for mandatory combined pharmacological and mechanical prophylaxis in high-risk critically ill patients. However, given the exploratory, non-predefined nature of these analyses and the small sample sizes, these findings should be considered hypothesis-generating. Future prospective studies with standardized sampling protocols and predefined outcome measures are needed to validate these biomarker associations. Recent work by Siddiqui et al. demonstrated that routine blood cellular indices have prognostic significance in PE, suggesting that simpler, widely available biomarkers could complement our findings (25, 35).

### 4.6 Fatal case analysis

The six fatal HA-VTE cases shared several features: advanced age (mean 79.9 years), male predominance (83%), infection/sepsis (5/6), multiple organ failure, and suboptimal or absent anticoagulation before VTE diagnosis. These findings echo those of Ma et al. (26) and Choffat et al. (27), who demonstrated that infection-induced hypercoagulability and inadequate prophylaxis synergistically increase mortality. Notably, three deaths were directly due to PTE, highlighting the need for earlier diagnosis and more aggressive intervention in high-risk patients. However, due to the very small number of events (*n* = 6), these observations are purely descriptive and should not be overinterpreted as robust predictors of mortality. Dubois-Silva et al. found that DVT symptoms were associated with higher 30-day mortality in acute PE, underscoring the importance of clinical recognition of VTE signs (28).

### 4.7 Strengths and limitations

The primary strengths of this study are its large, real-world sample size (393 HA-VTE cases over two years) and the inclusion of detailed procedural, biomarker, and departmental data. However, several limitations must be acknowledged. First, the retrospective design precludes causal inference and may introduce selection bias. Second, diagnostic practices for VTE (e.g., routine vs. symptom-triggered imaging) varied across departments, potentially leading to underascertainment of asymptomatic events. Third, the single-center nature limits generalizability to other regions or healthcare systems. Fourth, the absence of a validation cohort means that our risk factor estimates may be overfitted. Fifth, we did not collect data on race/ethnicity, genetic thrombophilias, or long-term outcomes beyond hospitalization. Sixth, the biomarker analyses were exploratory and not predefined, increasing the risk of type I error. Finally, the small number of deaths (*n* = 6) precludes any meaningful multivariable analysis of mortality predictors. Prospective, multi-center studies with standardized screening protocols and long-term follow-up are warranted to validate and extend our findings.

## 5 Implications for clinical practice and future research

Despite these limitations, our results have several actionable implications. First, hospitals should mandate universal VTE risk assessment (e.g., Caprini or Padua scores) at admission and postoperatively, integrated into electronic health records (29, 30). Second, targeted prophylaxis protocols should be enforced: low-molecular-weight heparin (e.g., enoxaparin 40 mg daily) or direct oral anticoagulants for high-risk surgical and cancer patients without bleeding risk, combined with intermittent pneumatic compression for all immobilized patients in ICUs and neurosurgery/orthopedics wards (31–33). Third, given the 43.28% prophylaxis implementation rate in one of our general hospitals, educational interventions for both physicians and nurses—similar to those successfully tested by Watt et al. (14) and Oh et al. (15)—are urgently needed. Fourth, for patients with persistent D-dimer elevation or sudden unexplained hypoxemia, immediate investigation for PTE should be triggered (22). Finally, infection control and early goal-directed therapy for sepsis may reduce hypercoagulability and secondary VTE risk (27). Future research should focus on dynamic risk models incorporating real-time biomarker monitoring, as suggested by machine learning approaches (e.g., Mora et al.) (34), and on context-adapted prophylaxis protocols validated in Eastern populations.

## 6 Conclusion

This study highlighted a strong association between specific surgical clusters-prolonged mechanical ventilation (≥96 h), central venous access, major orthopedic procedures (hip replacement, fracture fixation), and neurosurgical interventions (craniectomy, hematoma evacuation)—and HA-VTE incidence in a large Eastern Chinese tertiary medical center. The overall incidence was 0.87 per 1,000 admissions. The predominance of isolated DVT (78.4%) highlights the need for targeted lower-extremity surveillance in immobilized patients, while the 1.53% mortality—with half directly attributable to PE—underscores the clinical consequences of under-prophylaxis. Mechanistically, elevated D-dimer/fibrinogen, TF-bearing microparticles, and cancer procoagulant were directly detected in high-risk subgroups, confirming the relevance of endothelial injury, stasis, and hypercoagulability. Fatal cases were characterized by advanced age, multiple comorbidities, infection, iatrogenic procedures, and inadequate anticoagulation. Clinicians must strengthen VTE risk assessment and stratified management, with particular attention to postoperative, infected, and critically ill patients. Future efforts should focus on dynamic risk models, real-time biomarker monitoring, and standardized, context-adapted prophylaxis protocols to mitigate the HA-VTE burden.

## Data Availability

The raw data supporting the conclusions of this article will be made available by the authors, without undue reservation.
